# Supplementary material for: Pyruvate Dehydrogenase Kinase 1 inhibition mediated oxidative phosphorylation enhancement in cartilage promotes osteoarthritis progression
Source: BMC Musculoskelet Disord. 2023 Jul 20;24:597. doi: 10.1186/s12891-023-06585-6 (PMC10357736; doi:10.1186/s12891-023-06585-6)
Supplement: Supplementary file 1 — Additional file 1. [file 12891_2023_6585_MOESM1_ESM.zip › BP.Gsea.1653623667859/GOBP_ELECTRON_TRANSPORT_CHAIN.html]

Details for gene set GOBP\_ELECTRON\_TRANSPORT\_CHAIN[GSEA]

|  || Dataset | 55457\_collapsed\_to\_symbols.cls.cls #OA\_versus\_normal.cls.cls #OA\_versus\_normal\_repos |
| Phenotype | cls.cls#OA\_versus\_normal\_repos |
| Upregulated in class | OA |
| GeneSet | GOBP\_ELECTRON\_TRANSPORT\_CHAIN |
| Enrichment Score (ES) | 0.51531416 |
| Normalized Enrichment Score (NES) | 2.5296319 |
| Nominal p-value | 0.0 |
| FDR q-value | 0.0 |
| FWER p-Value | 0.0 |
Table: GSEA Results Summary

  

Fig 1: Enrichment plot: GOBP\_ELECTRON\_TRANSPORT\_CHAIN      
 Profile of the Running ES Score & Positions of GeneSet Members on the Rank Ordered List

  

| SYMBOL | TITLE | RANK IN GENE LIST | RANK METRIC SCORE | RUNNING ES | CORE ENRICHMENT || 1 | UQCR10 | "ubiquinol-cytochrome c reductase, complex III subunit X [Source:HGNC Symbol;Acc:HGNC:30863]" | 53 | 0.987 | 0.0160 | Yes |
| 2 | NDUFA8 | NADH:ubiquinone oxidoreductase subunit A8 [Source:HGNC Symbol;Acc:HGNC:7692] | 67 | 0.961 | 0.0347 | Yes |
| 3 | DGUOK | deoxyguanosine kinase [Source:HGNC Symbol;Acc:HGNC:2858] | 96 | 0.917 | 0.0513 | Yes |
| 4 | QDPR | quinoid dihydropteridine reductase [Source:HGNC Symbol;Acc:HGNC:9752] | 112 | 0.897 | 0.0684 | Yes |
| 5 | NDUFS3 | NADH:ubiquinone oxidoreductase core subunit S3 [Source:HGNC Symbol;Acc:HGNC:7710] | 177 | 0.836 | 0.0805 | Yes |
| 6 | NDUFA1 | NADH:ubiquinone oxidoreductase subunit A1 [Source:HGNC Symbol;Acc:HGNC:7683] | 194 | 0.826 | 0.0962 | Yes |
| 7 | GHITM | growth hormone inducible transmembrane protein [Source:HGNC Symbol;Acc:HGNC:17281] | 213 | 0.809 | 0.1113 | Yes |
| 8 | NDUFA13 | NADH:ubiquinone oxidoreductase subunit A13 [Source:HGNC Symbol;Acc:HGNC:17194] | 236 | 0.795 | 0.1258 | Yes |
| 9 | NDUFA10 | NADH:ubiquinone oxidoreductase subunit A10 [Source:HGNC Symbol;Acc:HGNC:7684] | 243 | 0.788 | 0.1415 | Yes |
| 10 | P4HA2 | prolyl 4-hydroxylase subunit alpha 2 [Source:HGNC Symbol;Acc:HGNC:8547] | 258 | 0.779 | 0.1564 | Yes |
| 11 | NDUFB6 | NADH:ubiquinone oxidoreductase subunit B6 [Source:HGNC Symbol;Acc:HGNC:7701] | 262 | 0.779 | 0.1720 | Yes |
| 12 | PDIA5 | protein disulfide isomerase family A member 5 [Source:HGNC Symbol;Acc:HGNC:24811] | 324 | 0.735 | 0.1823 | Yes |
| 13 | NDUFB3 | NADH:ubiquinone oxidoreductase subunit B3 [Source:HGNC Symbol;Acc:HGNC:7698] | 483 | 0.667 | 0.1836 | Yes |
| 14 | CYBA | cytochrome b-245 alpha chain [Source:HGNC Symbol;Acc:HGNC:2577] | 501 | 0.658 | 0.1957 | Yes |
| 15 | NDUFS4 | NADH:ubiquinone oxidoreductase subunit S4 [Source:HGNC Symbol;Acc:HGNC:7711] | 531 | 0.647 | 0.2067 | Yes |
| 16 | NDUFA3 | NADH:ubiquinone oxidoreductase subunit A3 [Source:HGNC Symbol;Acc:HGNC:7686] | 550 | 0.641 | 0.2184 | Yes |
| 17 | COQ9 | coenzyme Q9 [Source:HGNC Symbol;Acc:HGNC:25302] | 561 | 0.636 | 0.2306 | Yes |
| 18 | DLD | dihydrolipoamide dehydrogenase [Source:HGNC Symbol;Acc:HGNC:2898] | 565 | 0.635 | 0.2434 | Yes |
| 19 | COX5B | cytochrome c oxidase subunit 5B [Source:HGNC Symbol;Acc:HGNC:2269] | 585 | 0.628 | 0.2547 | Yes |
| 20 | NDUFA4 | NDUFA4 mitochondrial complex associated [Source:HGNC Symbol;Acc:HGNC:7687] | 624 | 0.613 | 0.2643 | Yes |
| 21 | COX6A1 | cytochrome c oxidase subunit 6A1 [Source:HGNC Symbol;Acc:HGNC:2277] | 653 | 0.605 | 0.2745 | Yes |
| 22 | NDUFA2 | NADH:ubiquinone oxidoreductase subunit A2 [Source:HGNC Symbol;Acc:HGNC:7685] | 878 | 0.549 | 0.2682 | Yes |
| 23 | NDUFB5 | NADH:ubiquinone oxidoreductase subunit B5 [Source:HGNC Symbol;Acc:HGNC:7700] | 902 | 0.542 | 0.2775 | Yes |
| 24 | NDUFC2 | NADH:ubiquinone oxidoreductase subunit C2 [Source:HGNC Symbol;Acc:HGNC:7706] | 929 | 0.532 | 0.2863 | Yes |
| 25 | NDUFA9 | NADH:ubiquinone oxidoreductase subunit A9 [Source:HGNC Symbol;Acc:HGNC:7693] | 943 | 0.529 | 0.2962 | Yes |
| 26 | DNAJC15 | DnaJ heat shock protein family (Hsp40) member C15 [Source:HGNC Symbol;Acc:HGNC:20325] | 954 | 0.527 | 0.3062 | Yes |
| 27 | NDUFB4 | NADH:ubiquinone oxidoreductase subunit B4 [Source:HGNC Symbol;Acc:HGNC:7699] | 968 | 0.525 | 0.3159 | Yes |
| 28 | UQCRQ | ubiquinol-cytochrome c reductase complex III subunit VII [Source:HGNC Symbol;Acc:HGNC:29594] | 986 | 0.521 | 0.3252 | Yes |
| 29 | ALDH2 | aldehyde dehydrogenase 2 family member [Source:HGNC Symbol;Acc:HGNC:404] | 1003 | 0.517 | 0.3345 | Yes |
| 30 | NDUFB7 | NADH:ubiquinone oxidoreductase subunit B7 [Source:HGNC Symbol;Acc:HGNC:7702] | 1033 | 0.510 | 0.3427 | Yes |
| 31 | NDUFB1 | NADH:ubiquinone oxidoreductase subunit B1 [Source:HGNC Symbol;Acc:HGNC:7695] | 1055 | 0.506 | 0.3514 | Yes |
| 32 | NDUFS7 | NADH:ubiquinone oxidoreductase core subunit S7 [Source:HGNC Symbol;Acc:HGNC:7714] | 1071 | 0.501 | 0.3605 | Yes |
| 33 | NDUFC1 | NADH:ubiquinone oxidoreductase subunit C1 [Source:HGNC Symbol;Acc:HGNC:7705] | 1091 | 0.499 | 0.3692 | Yes |
| 34 | NDUFA7 | NADH:ubiquinone oxidoreductase subunit A7 [Source:HGNC Symbol;Acc:HGNC:7691] | 1097 | 0.496 | 0.3790 | Yes |
| 35 | FDX1 | ferredoxin 1 [Source:HGNC Symbol;Acc:HGNC:3638] | 1168 | 0.483 | 0.3834 | Yes |
| 36 | NDUFS8 | NADH:ubiquinone oxidoreductase core subunit S8 [Source:HGNC Symbol;Acc:HGNC:7715] | 1208 | 0.474 | 0.3900 | Yes |
| 37 | NDUFS1 | NADH:ubiquinone oxidoreductase core subunit S1 [Source:HGNC Symbol;Acc:HGNC:7707] | 1229 | 0.471 | 0.3981 | Yes |
| 38 | ETFB | electron transfer flavoprotein subunit beta [Source:HGNC Symbol;Acc:HGNC:3482] | 1289 | 0.460 | 0.4029 | Yes |
| 39 | PARK7 | Parkinsonism associated deglycase [Source:HGNC Symbol;Acc:HGNC:16369] | 1406 | 0.442 | 0.4028 | Yes |
| 40 | NDUFS6 | NADH:ubiquinone oxidoreductase subunit S6 [Source:HGNC Symbol;Acc:HGNC:7713] | 1417 | 0.441 | 0.4111 | Yes |
| 41 | CYB561 | cytochrome b561 [Source:HGNC Symbol;Acc:HGNC:2571] | 1459 | 0.435 | 0.4167 | Yes |
| 42 | ISCU | iron-sulfur cluster assembly enzyme [Source:HGNC Symbol;Acc:HGNC:29882] | 1530 | 0.423 | 0.4199 | Yes |
| 43 | NOX4 | NADPH oxidase 4 [Source:HGNC Symbol;Acc:HGNC:7891] | 1563 | 0.418 | 0.4260 | Yes |
| 44 | NQO2 | N-ribosyldihydronicotinamide:quinone reductase 2 [Source:HGNC Symbol;Acc:HGNC:7856] | 1568 | 0.418 | 0.4342 | Yes |
| 45 | COX11 | cytochrome c oxidase copper chaperone COX11 [Source:HGNC Symbol;Acc:HGNC:2261] | 1622 | 0.410 | 0.4385 | Yes |
| 46 | NDUFV2 | NADH:ubiquinone oxidoreductase core subunit V2 [Source:HGNC Symbol;Acc:HGNC:7717] | 1642 | 0.407 | 0.4453 | Yes |
| 47 | NDUFV1 | NADH:ubiquinone oxidoreductase core subunit V1 [Source:HGNC Symbol;Acc:HGNC:7716] | 1663 | 0.404 | 0.4520 | Yes |
| 48 | ME1 | malic enzyme 1 [Source:HGNC Symbol;Acc:HGNC:6983] | 1746 | 0.392 | 0.4536 | Yes |
| 49 | ETFA | electron transfer flavoprotein subunit alpha [Source:HGNC Symbol;Acc:HGNC:3481] | 1797 | 0.385 | 0.4576 | Yes |
| 50 | SNCA | synuclein alpha [Source:HGNC Symbol;Acc:HGNC:11138] | 1800 | 0.384 | 0.4653 | Yes |
| 51 | SDHB | succinate dehydrogenase complex iron sulfur subunit B [Source:HGNC Symbol;Acc:HGNC:10681] | 1818 | 0.382 | 0.4717 | Yes |
| 52 | SURF1 | SURF1 cytochrome c oxidase assembly factor [Source:HGNC Symbol;Acc:HGNC:11474] | 1829 | 0.381 | 0.4788 | Yes |
| 53 | ME2 | malic enzyme 2 [Source:HGNC Symbol;Acc:HGNC:6984] | 1870 | 0.377 | 0.4833 | Yes |
| 54 | SRD5A1 | steroid 5 alpha-reductase 1 [Source:HGNC Symbol;Acc:HGNC:11284] | 1973 | 0.364 | 0.4828 | Yes |
| 55 | COX7A1 | cytochrome c oxidase subunit 7A1 [Source:HGNC Symbol;Acc:HGNC:2287] | 2045 | 0.356 | 0.4845 | Yes |
| 56 | PINK1 | PTEN induced kinase 1 [Source:HGNC Symbol;Acc:HGNC:14581] | 2097 | 0.350 | 0.4877 | Yes |
| 57 | COX4I1 | cytochrome c oxidase subunit 4I1 [Source:HGNC Symbol;Acc:HGNC:2265] | 2167 | 0.342 | 0.4893 | Yes |
| 58 | MAOB | monoamine oxidase B [Source:HGNC Symbol;Acc:HGNC:6834] | 2192 | 0.339 | 0.4943 | Yes |
| 59 | NCF1 | neutrophil cytosolic factor 1 [Source:HGNC Symbol;Acc:HGNC:7660] | 2195 | 0.339 | 0.5011 | Yes |
| 60 | SDHA | succinate dehydrogenase complex flavoprotein subunit A [Source:HGNC Symbol;Acc:HGNC:10680] | 2231 | 0.335 | 0.5052 | Yes |
| 61 | NDUFS5 | NADH:ubiquinone oxidoreductase subunit S5 [Source:HGNC Symbol;Acc:HGNC:7712] | 2267 | 0.330 | 0.5092 | Yes |
| 62 | CCNB1 | cyclin B1 [Source:HGNC Symbol;Acc:HGNC:1579] | 2359 | 0.319 | 0.5087 | Yes |
| 63 | CHCHD2 | coiled-coil-helix-coiled-coil-helix domain containing 2 [Source:HGNC Symbol;Acc:HGNC:21645] | 2431 | 0.310 | 0.5094 | Yes |
| 64 | NDUFB2 | NADH:ubiquinone oxidoreductase subunit B2 [Source:HGNC Symbol;Acc:HGNC:7697] | 2460 | 0.307 | 0.5135 | Yes |
| 65 | COX7A2L | cytochrome c oxidase subunit 7A2 like [Source:HGNC Symbol;Acc:HGNC:2289] | 2517 | 0.302 | 0.5153 | Yes |
| 66 | NDUFB8 | NADH:ubiquinone oxidoreductase subunit B8 [Source:HGNC Symbol;Acc:HGNC:7703] | 2623 | 0.288 | 0.5130 | No |
| 67 | AKR7A2 | aldo-keto reductase family 7 member A2 [Source:HGNC Symbol;Acc:HGNC:389] | 2690 | 0.280 | 0.5135 | No |
| 68 | HSD17B6 | hydroxysteroid 17-beta dehydrogenase 6 [Source:HGNC Symbol;Acc:HGNC:23316] | 2965 | 0.254 | 0.4973 | No |
| 69 | NDUFS2 | NADH:ubiquinone oxidoreductase core subunit S2 [Source:HGNC Symbol;Acc:HGNC:7708] | 3072 | 0.243 | 0.4939 | No |
| 70 | AKR1B1 | aldo-keto reductase family 1 member B [Source:HGNC Symbol;Acc:HGNC:381] | 3138 | 0.236 | 0.4937 | No |
| 71 | SDHC | succinate dehydrogenase complex subunit C [Source:HGNC Symbol;Acc:HGNC:10682] | 3145 | 0.235 | 0.4980 | No |
| 72 | HAAO | "3-hydroxyanthranilate 3,4-dioxygenase [Source:HGNC Symbol;Acc:HGNC:4796]" | 3209 | 0.229 | 0.4978 | No |
| 73 | GBA | glucosylceramidase beta [Source:HGNC Symbol;Acc:HGNC:4177] | 3327 | 0.219 | 0.4931 | No |
| 74 | COX15 | cytochrome c oxidase assembly homolog COX15 [Source:HGNC Symbol;Acc:HGNC:2263] | 3727 | 0.183 | 0.4656 | No |
| 75 | CDK1 | cyclin dependent kinase 1 [Source:HGNC Symbol;Acc:HGNC:1722] | 3744 | 0.182 | 0.4681 | No |
| 76 | ENOX2 | ecto-NOX disulfide-thiol exchanger 2 [Source:HGNC Symbol;Acc:HGNC:2259] | 3769 | 0.179 | 0.4699 | No |
| 77 | UQCRH | ubiquinol-cytochrome c reductase hinge protein [Source:HGNC Symbol;Acc:HGNC:12590] | 3827 | 0.174 | 0.4689 | No |
| 78 | NDUFA6 | NADH:ubiquinone oxidoreductase subunit A6 [Source:HGNC Symbol;Acc:HGNC:7690] | 3834 | 0.173 | 0.4720 | No |
| 79 | GLDC | glycine decarboxylase [Source:HGNC Symbol;Acc:HGNC:4313] | 3913 | 0.163 | 0.4692 | No |
| 80 | SDHD | succinate dehydrogenase complex subunit D [Source:HGNC Symbol;Acc:HGNC:10683] | 4055 | 0.151 | 0.4613 | No |
| 81 | CIAPIN1 | cytokine induced apoptosis inhibitor 1 [Source:HGNC Symbol;Acc:HGNC:28050] | 4161 | 0.141 | 0.4559 | No |
| 82 | NDUFAF1 | NADH:ubiquinone oxidoreductase complex assembly factor 1 [Source:HGNC Symbol;Acc:HGNC:18828] | 4388 | 0.122 | 0.4407 | No |
| 83 | NDUFAB1 | NADH:ubiquinone oxidoreductase subunit AB1 [Source:HGNC Symbol;Acc:HGNC:7694] | 4445 | 0.118 | 0.4388 | No |
| 84 | COX7C | cytochrome c oxidase subunit 7C [Source:HGNC Symbol;Acc:HGNC:2292] | 4539 | 0.110 | 0.4337 | No |
| 85 | GSR | glutathione-disulfide reductase [Source:HGNC Symbol;Acc:HGNC:4623] | 4597 | 0.105 | 0.4314 | No |
| 86 | ADH5 | "alcohol dehydrogenase 5 (class III), chi polypeptide [Source:HGNC Symbol;Acc:HGNC:253]" | 4720 | 0.094 | 0.4238 | No |
| 87 | DHRS3 | dehydrogenase/reductase 3 [Source:HGNC Symbol;Acc:HGNC:17693] | 4770 | 0.089 | 0.4218 | No |
| 88 | CYC1 | cytochrome c1 [Source:HGNC Symbol;Acc:HGNC:2579] | 4879 | 0.082 | 0.4150 | No |
| 89 | UQCRB | ubiquinol-cytochrome c reductase binding protein [Source:HGNC Symbol;Acc:HGNC:12582] | 5003 | 0.073 | 0.4068 | No |
| 90 | AKR7A3 | aldo-keto reductase family 7 member A3 [Source:HGNC Symbol;Acc:HGNC:390] | 5192 | 0.057 | 0.3933 | No |
| 91 | COX6A2 | cytochrome c oxidase subunit 6A2 [Source:HGNC Symbol;Acc:HGNC:2279] | 5242 | 0.053 | 0.3905 | No |
| 92 | COX7A2P2 | cytochrome c oxidase subunit 7A2 pseudogene 2 [Source:HGNC Symbol;Acc:HGNC:2290] | 5263 | 0.051 | 0.3900 | No |
| 93 | COX5A | cytochrome c oxidase subunit 5A [Source:HGNC Symbol;Acc:HGNC:2267] | 5329 | 0.045 | 0.3858 | No |
| 94 | SLC25A12 | solute carrier family 25 member 12 [Source:HGNC Symbol;Acc:HGNC:10982] | 5506 | 0.032 | 0.3727 | No |
| 95 | UQCRFS1 | "ubiquinol-cytochrome c reductase, Rieske iron-sulfur polypeptide 1 [Source:HGNC Symbol;Acc:HGNC:12587]" | 5509 | 0.032 | 0.3732 | No |
| 96 | BID | BH3 interacting domain death agonist [Source:HGNC Symbol;Acc:HGNC:1050] | 5715 | 0.014 | 0.3574 | No |
| 97 | GPX2 | glutathione peroxidase 2 [Source:HGNC Symbol;Acc:HGNC:4554] | 5808 | 0.008 | 0.3504 | No |
| 98 | AKR1C4 | aldo-keto reductase family 1 member C4 [Source:HGNC Symbol;Acc:HGNC:387] | 5843 | 0.005 | 0.3478 | No |
| 99 | CYB5A | cytochrome b5 type A [Source:HGNC Symbol;Acc:HGNC:2570] | 5850 | 0.005 | 0.3475 | No |
| 100 | POLG2 | "DNA polymerase gamma 2, accessory subunit [Source:HGNC Symbol;Acc:HGNC:9180]" | 5892 | 0.003 | 0.3443 | No |
| 101 | GLRX2 | glutaredoxin 2 [Source:HGNC Symbol;Acc:HGNC:16065] | 6116 | -0.014 | 0.3271 | No |
| 102 | CYP19A1 | cytochrome P450 family 19 subfamily A member 1 [Source:HGNC Symbol;Acc:HGNC:2594] | 6388 | -0.034 | 0.3066 | No |
| 103 | CYBB | cytochrome b-245 beta chain [Source:HGNC Symbol;Acc:HGNC:2578] | 6610 | -0.050 | 0.2903 | No |
| 104 | NDUFA5 | NADH:ubiquinone oxidoreductase subunit A5 [Source:HGNC Symbol;Acc:HGNC:7688] | 6789 | -0.062 | 0.2776 | No |
| 105 | NCF2 | neutrophil cytosolic factor 2 [Source:HGNC Symbol;Acc:HGNC:7661] | 6871 | -0.069 | 0.2727 | No |
| 106 | GPD2 | glycerol-3-phosphate dehydrogenase 2 [Source:HGNC Symbol;Acc:HGNC:4456] | 7206 | -0.097 | 0.2485 | No |
| 107 | GFUS | GDP-L-fucose synthase [Source:HGNC Symbol;Acc:HGNC:12390] | 7317 | -0.103 | 0.2420 | No |
| 108 | GPD1 | glycerol-3-phosphate dehydrogenase 1 [Source:HGNC Symbol;Acc:HGNC:4455] | 7420 | -0.112 | 0.2363 | No |
| 109 | ASPH | aspartate beta-hydroxylase [Source:HGNC Symbol;Acc:HGNC:757] | 7533 | -0.121 | 0.2300 | No |
| 110 | LOXL2 | lysyl oxidase like 2 [Source:HGNC Symbol;Acc:HGNC:6666] | 7675 | -0.133 | 0.2217 | No |
| 111 | IDO1 | "indoleamine 2,3-dioxygenase 1 [Source:HGNC Symbol;Acc:HGNC:6059]" | 7782 | -0.140 | 0.2163 | No |
| 112 | NDOR1 | NADPH dependent diflavin oxidoreductase 1 [Source:HGNC Symbol;Acc:HGNC:29838] | 7982 | -0.154 | 0.2038 | No |
| 113 | CYCS | "cytochrome c, somatic [Source:HGNC Symbol;Acc:HGNC:19986]" | 8410 | -0.186 | 0.1742 | No |
| 114 | SLC25A22 | solute carrier family 25 member 22 [Source:HGNC Symbol;Acc:HGNC:19954] | 8682 | -0.208 | 0.1572 | No |
| 115 | SLC25A13 | solute carrier family 25 member 13 [Source:HGNC Symbol;Acc:HGNC:10983] | 8713 | -0.210 | 0.1592 | No |
| 116 | DEGS1 | "delta 4-desaturase, sphingolipid 1 [Source:HGNC Symbol;Acc:HGNC:13709]" | 8885 | -0.223 | 0.1503 | No |
| 117 | PHGDH | phosphoglycerate dehydrogenase [Source:HGNC Symbol;Acc:HGNC:8923] | 9245 | -0.252 | 0.1274 | No |
| 118 | DHRS2 | dehydrogenase/reductase 2 [Source:HGNC Symbol;Acc:HGNC:18349] | 9363 | -0.261 | 0.1235 | No |
| 119 | RDH16 | retinol dehydrogenase 16 [Source:HGNC Symbol;Acc:HGNC:29674] | 9900 | -0.312 | 0.0879 | No |
| 120 | POR | cytochrome p450 oxidoreductase [Source:HGNC Symbol;Acc:HGNC:9208] | 10178 | -0.338 | 0.0732 | No |
| 121 | ETFDH | electron transfer flavoprotein dehydrogenase [Source:HGNC Symbol;Acc:HGNC:3483] | 10203 | -0.341 | 0.0783 | No |
| 122 | TAFAZZIN | "tafazzin, phospholipid-lysophospholipid transacylase [Source:HGNC Symbol;Acc:HGNC:11577]" | 10429 | -0.364 | 0.0681 | No |
| 123 | PPARGC1A | PPARG coactivator 1 alpha [Source:HGNC Symbol;Acc:HGNC:9237] | 10504 | -0.374 | 0.0699 | No |
| 124 | PLEC | plectin [Source:HGNC Symbol;Acc:HGNC:9069] | 10799 | -0.405 | 0.0552 | No |
| 125 | MYBBP1A | MYB binding protein 1a [Source:HGNC Symbol;Acc:HGNC:7546] | 11188 | -0.456 | 0.0341 | No |
| 126 | IDH3B | isocitrate dehydrogenase (NAD(+)) 3 non-catalytic subunit beta [Source:HGNC Symbol;Acc:HGNC:5385] | 11304 | -0.472 | 0.0348 | No |
| 127 | STEAP4 | STEAP4 metalloreductase [Source:HGNC Symbol;Acc:HGNC:21923] | 11340 | -0.477 | 0.0418 | No |
| 128 | SCO2 | synthesis of cytochrome C oxidase 2 [Source:HGNC Symbol;Acc:HGNC:10604] | 11410 | -0.488 | 0.0464 | No |
| 129 | AOC2 | amine oxidase copper containing 2 [Source:HGNC Symbol;Acc:HGNC:549] | 11550 | -0.508 | 0.0459 | No |
| 130 | CYP1A2 | cytochrome P450 family 1 subfamily A member 2 [Source:HGNC Symbol;Acc:HGNC:2596] | 11719 | -0.536 | 0.0437 | No |
| 131 | ALDH4A1 | aldehyde dehydrogenase 4 family member A1 [Source:HGNC Symbol;Acc:HGNC:406] | 12080 | -0.614 | 0.0281 | No |
| 132 | SOD2 | superoxide dismutase 2 [Source:HGNC Symbol;Acc:HGNC:11180] | 12685 | -0.853 | -0.0018 | No |
| 133 | MT-ND5 | mitochondrially encoded NADH:ubiquinone oxidoreductase core subunit 5 [Source:HGNC Symbol;Acc:HGNC:7461] | 12743 | -0.906 | 0.0123 | No |
Table: GSEA details [plain text format]

  

Fig 2: GOBP\_ELECTRON\_TRANSPORT\_CHAIN      
 Blue-Pink O' Gram in the Space of the Analyzed GeneSet

  

Fig 3: GOBP\_ELECTRON\_TRANSPORT\_CHAIN: Random ES distribution      
 Gene set null distribution of ES for **GOBP\_ELECTRON\_TRANSPORT\_CHAIN**
